# A Case Report of a 36-year-old Male Diagnosed with a Spontaneous Coronary Artery Dissection

**DOI:** 10.5070/M5.52022

**Published:** 2025-01-31

**Authors:** Stephen DeWitt, Jacob McClinton, Daniel Jarrell

**Affiliations:** * Memorial Health System, Marietta Memorial Hospital Department of Emergency Medicine, Marietta, OH

## Abstract

This case report discusses a 36-year-old male who presented to the emergency department with an atypical story for acute coronary syndrome (ACS). Initially, the patient was felt to have a non-diagnostic electrocardiogram (ECG). Once the laboratory test results were obtained and the initial high-sensitivity troponin was noted to be elevated, the initial ECG was again reviewed. The patient was felt this time to have an abnormal ECG, demonstrating borderline ST elevation in leads I, aVL, and V2–V5. The interventional cardiologist on call was contacted, and the cardiac catheterization lab was activated. Upon left heart catheterization, the patient was found to have a spontaneous coronary artery dissection (SCAD) of the distal left anterior descending (LAD) artery. Post-catheterization, the patient was observed on cardiac telemetry and started on dual antiplatelet therapy. Echocardiogram revealed a preserved ejection fraction (EF), but hypokinesis of the apical anterior, anterolateral, inferior, and apical myocardium. The patient was discharged within 48 hours without any complications.

**Topics:**

Electrocardiogram, ECG, cardiology, acute coronary syndrome, spontaneous coronary artery dissection, SCAD.

**Figure:**
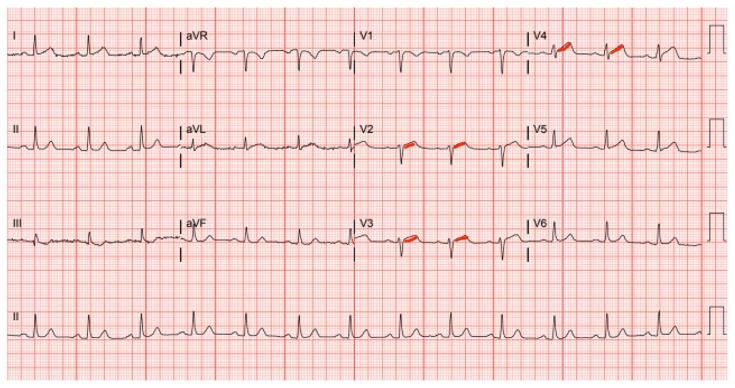


**Figure:**
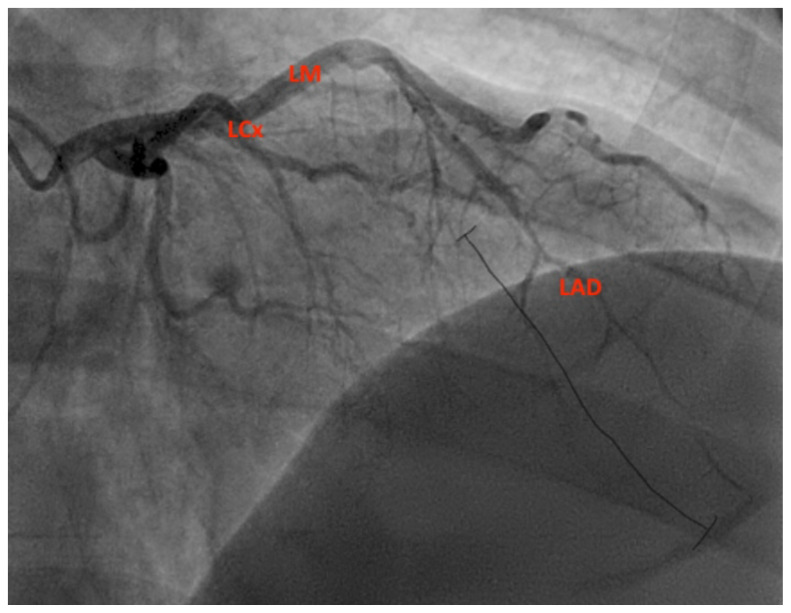


## Brief introduction

Spontaneous coronary artery dissection (SCAD) is the nontraumatic, noniatrogenic separation of the wall of a coronary artery, forming a false lumen. This separation or tear occurs in the tunica intima and results in a false lumen and intramural hematoma. This hematoma may expand to compress the true lumen and subsequently obstruct coronary blood flow, leading to an acute myocardial infarction (MI) of the downstream cardiac tissue.^1^–^5^ Spontaneous coronary artery dissection is a cause of ACS which has an unknown prevalence, given the fact that it is likely underdiagnosed.^1^–^4^ The incidence seems to be increasing in recent years as it becomes more recognized as a cause of ACS due to increasingly advanced imaging techniques and aggressive early percutaneous coronary intervention (PCI).^1^,^3^ Spontaneous coronary artery dissection may be responsible for up to 4% of all ACS cases.^1^ Patients with SCAD often lack traditional cardiovascular risk factors; therefore, it is important to consider as a cause of ACS, particularly in younger patients.

Spontaneous coronary artery dissection is more common in females. It is thought to be associated with non-traditional risk factors for heart disease, including pregnancy, hormonal changes, migraines,, environmental stressors, and inherited arteriopathies (fibromuscular dysplasia, Marfan, Ehlers-Danlos).^1^–^4^,^6^–^8^ Up to 2.5% of fibromuscular dysplasia (FMD) patients will experience SCAD, and it is the most common cause of myocardial infarction in pregnant women.^5^,^8^ There is a significant female predominance, with women accounting for up to 92% of cases of SCAD.^1^,^3^ Women affected by SCAD can range in age from teens to women in their 80s, with a mean age of 50 years old,^1^–^3^ and SCAD may account for up to 35% of ACS cases in women under 50 years old.^1^ Data for men is more limited, given the significant female predominance. In male patients, SCAD has a slightly younger mean age at 49 years old. In SCAD patients with pre-existing coronary artery disease (CAD), males are predominant^1^,^4^ and males have a 3.5-fold higher risk of mortality from SCAD.^9^

## Presenting concerns and clinical findings

The patient was a 36-year-old male who presented to the emergency department via emergency medical services (EMS) at 11:13 a.m. with a complaint of chest discomfort that he described as a “bubble in my chest” that started around 10:00 a.m. while he was eating breakfast. He experienced a sensation of flushing in his face and an unusual discomfort in his bilateral shoulders. During transport, the patient experienced a transient episode of shortness of breath that had since passed. On arrival to the ED, he reportedly felt much better than he did previously, but continued to have the sensation of a substernal bubble. The patient reported recently having three days of rhinorrhea and non-productive cough without fever. There was no chest pain or discomfort prior to the morning of emergency department presentation. He did have a past smoking history but transitioned from traditional cigarettes to electronic cigarettes about two years prior. He denied a history of hypercholesterolemia, hypertension, previous cardiac disease, recent surgery, prolonged immobilization, malignancy, or history of venous thromboembolism. He does have a family history of CAD in his father. Review of systems was negative for any lower extremity edema, palpitations, claudication, dizziness, or syncope.

The patient’s vital signs were stable on arrival with a blood pressure of 134/72, heart rate of 84, respiratory rate of 18, oxygen saturation of 98% on room air, and temperature of 99.0ºF. Physical exam revealed an alert and oriented male in no acute distress with a regular rate and rhythm and normal heart sounds.

## Significant findings

The initial ECG obtained from the patient shows subtle ST-segment elevation noted in leads I, aVL, and V2–V5, suggestive of pathology of the left anterior descending artery.

Laboratory studies were remarkable for an initial high-sensitivity troponin of 51ng/L, which corresponds to a traditional troponin of 0.051ng/dL. In our lab, a troponin of 23 is the upper 99^th^ percentile for men, and greater than or equal to 52 is consistent with myocardial infarction in a patient presenting with chest pain and no other cause for an elevated troponin. Repeat troponin measurements were ordered in the emergency department but were cancelled because the patient went for catheterization after the first measurement.

## Patient course

Given the concerning ECG and troponin findings, interventional cardiology was consulted; the patient was given a bolus of heparin and was taken for emergent cardiac catheterization. With the patient’s preceding viral URI symptoms, interventional cardiology considered pericarditis to be a more likely cause for the ECG changes rather than an ischemic event. Interventional cardiology was contacted only once, and after initial consultation, the patient was taken to the cardiac catheterization lab. The results of the catheterization revealed a spontaneous coronary artery dissection of the distal portion of the left anterior descending coronary artery, which can be seen in the image of the angiogram, with the diseased portion notated between the brackets. The left main coronary (LM) and left circumflex artery (LCx) are also labeled.

The patient underwent an echocardiogram while in the hospital, which revealed a preserved ejection fraction, but severe hypokinesis of the apical anterior, anterolateral, inferior, and apical myocardium. After approximately 24 hours of cardiac observation, the patient was discharged from the hospital with an order for an outpatient computed tomography angiogram (CTA) of the thoracic aorta to further investigate for underlying FMD.

The patient presented to his cardiologist’s office for follow-up 10 days after discharge and was doing well at that time. He had ceased his electronic cigarette use. His low-density lipoprotein (LDL) was at goal at 95. He was also requesting to return to work with light-duty precautions, which was granted to him. A CTA was ordered and obtained several days later. The CTA did not reveal any underlying abnormalities and was interpreted as normal.

## Discussion

In otherwise young and relatively healthy patients who present with chest pain and ECG changes, SCAD must remain on the differential, especially in the absence of traditional cardiac risk factors. Our patient had a smoking history but was unaware of any additional risk factors outside of CAD in his father. His story, while exhibiting symptoms which can be associated with ACS, was largely atypical. Our patient also did not have typical risk factors for SCAD because he was a male with no known history of arteriopathies such as FMD, Marfan, or Ehlers-Danlos.

Spontaneous coronary artery dissection is likely an underdiagnosed and underrecognized cause of ACS, particularly in young, healthy individuals. These patients lack traditional ACS risk factors, and presentations can be subtle or variable in a way that is indistinguishable from other causes of chest pain.^1^–^4^ The mechanism of myocardial injury is coronary obstruction caused by an intramural hematoma or intimal disruption rather than an intraluminal thrombus or atherosclerotic plaque rupture.^1^ The classic SCAD clinical presentation includes chest pain or chest pain equivalent symptoms with elevated biomarkers, with or without associated ST-segment elevation. Of note, SCAD onset is often associated with a recent history of extreme physical or emotional distress^1^–^2^.

Complications associated with SCAD are common. This includes recurrent SCAD often associated with MI, congestive heart failure (CHF), stroke, and death.^1^ For this reason it is of utmost importance that SCAD remains on the differential and be identified. Risk of major adverse cardiovascular event (MACE) in the intermediate-term follow-up period of two to three years was found to be 10–30% with recurrent MI from recurrent SCAD in 15–22% of patients. The long-term follow-up period of 4–10 years is associated with 30% risk of SCAD recurrence.^1^ Severe coronary tortuosity, often associated with FMD, is a known risk factor for recurrence, and smoking was associated with a 15-fold increase in mortality from SCAD.^1^,^9^

Pregnant patients with SCAD tend to have poorer prognoses, and present in the postpartum period in 73% of cases, and 76% of these patients will present with ST-elevation myocardial infarction (STEMI). Complications are common in pregnant patients, including cardiogenic shock (24%) and ventricular fibrillation requiring defibrillation (16%), with 28% requiring mechanical cardiovascular support.^1^,^2^ Although SCAD has gained larger recognition and attention over the past decade, there continues to be no consensus on the preferred initial management in these patients^1^–^2^. Percutaneous coronary intervention is a risky option given the increased fragility of dissected vessels. Patients with active or ongoing ischemia or hemodynamic instability are most appropriate for PCI^1^–^2^. Repeat angiography or PCI is typically not performed unless benefits outweigh the risk of iatrogenic dissection^1^. Medical management includes antiplatelets, statins, and beta-blockers. While beta-blockers have demonstrated a decreased risk for recurrent SCAD, statins were found to have no association with recurrence. Antiplatelet therapy is recommended for SCAD patients who undergo PCI, but evidence for those patients who do not undergo PCI is lacking^1^–^2^. Coronary artery bypass grafting is an option in left main stem or proximal disease but is not generally recommended unless there is clinical worsening after conservative measures and/or PCI.^1^ Spontaneous coronary artery dissection has a 1% mortality risk, and 14% of patients will require revascularization due to extension of dissection. Review of several studies since 2008 demonstrated a 0–3% mortality risk post-revascularization.^1^,^9^

As more data and research are collected, treatment recommendations may be updated, and the incidence is likely to continue to increase. Emergency physicians should maintain a high index of suspicion for SCAD as the cause of chest pain when dealing with younger patients, even if they have no proven risk factors. From an emergency medicine standpoint, patients having active or persistent symptoms may benefit from repeat ECG and troponin measurements to better identify and screen for SCAD.

## Supplementary Information









## References

[1] Hayes SN, Kim ES, Saw J (2018). Spontaneous coronary artery dissection: Current state of the science: A scientific statement from the American Heart Association. Circulation.

[2] Hayes SN, Tweet MS, Adlam D (2020). Spontaneous coronary artery dissection. J Am Coll Cardiol.

[3] Tweet MS, Hayes SN, Pitta SR (2012). Clinical features, management, and prognosis of spontaneous coronary artery dissection. Circulation.

[4] Alfonso F, Paulo M, Lennie V (2012). Spontaneous coronary artery dissection. JACC: Cardiovasc Interv.

[5] Nepal S, Bishop MA (2025). Spontaneous coronary artery dissection. [Updated 2023 Jun 21]. StatPearls [Internet].

[6] Parekh JD, Chauhan S, Porter JL (2025). Coronary Artery Dissection (Archived) [Updated 2023 Jun 19]. StatPearls [Internet].

[7] Shahjehan RD, Sharma S, Bhutta BS (2025). Coronary Artery Disease. [Updated 2024 Oct 9]. StatPearls [Internet].

[8] Whelan LJ, Tintinalli JE, Ma O, Yealy DM (2020). Comorbid disorders in pregnancy. Tintinalli’s Emergency Medicine: A Comprehensive Study Guide.

[9] Adams C, He M, Hughes I, Singh K (2021). Mortality in spontaneous coronary artery dissection: A systematic review and meta-analysis. Catheter Cardiovasc Interv.

