# Supplementary figures and images for: A Case Report of a 36-year-old Male Diagnosed with a Spontaneous Coronary Artery Dissection
Source: J Educ Teach Emerg Med. 2025 Jan 31;11(1):V19–23. doi: 10.5070/M5.52022 (PMC12880892; doi:10.5070/M5.52022)

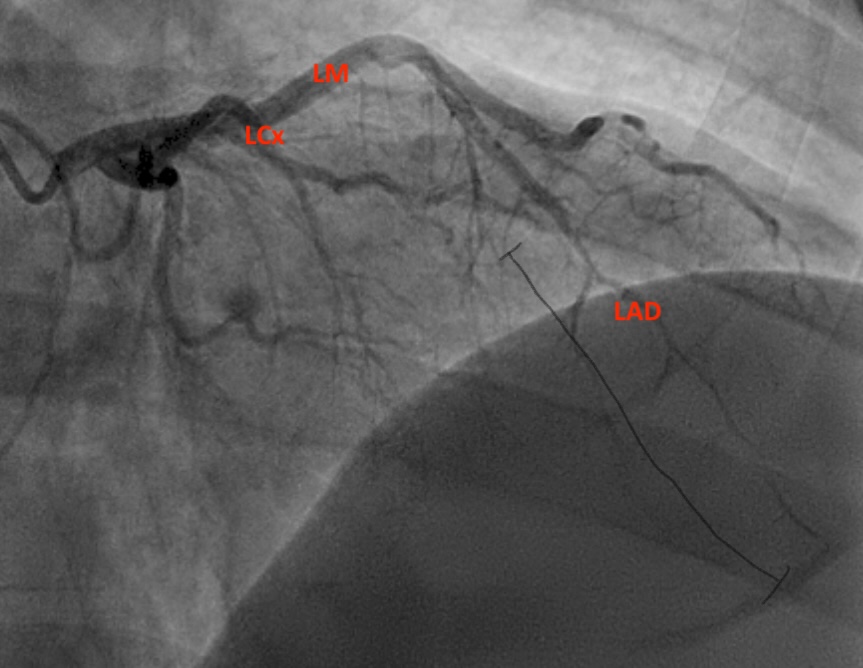

Supplement: Supplementary file 1 [file 11-1-V19-Supp1.jpg]

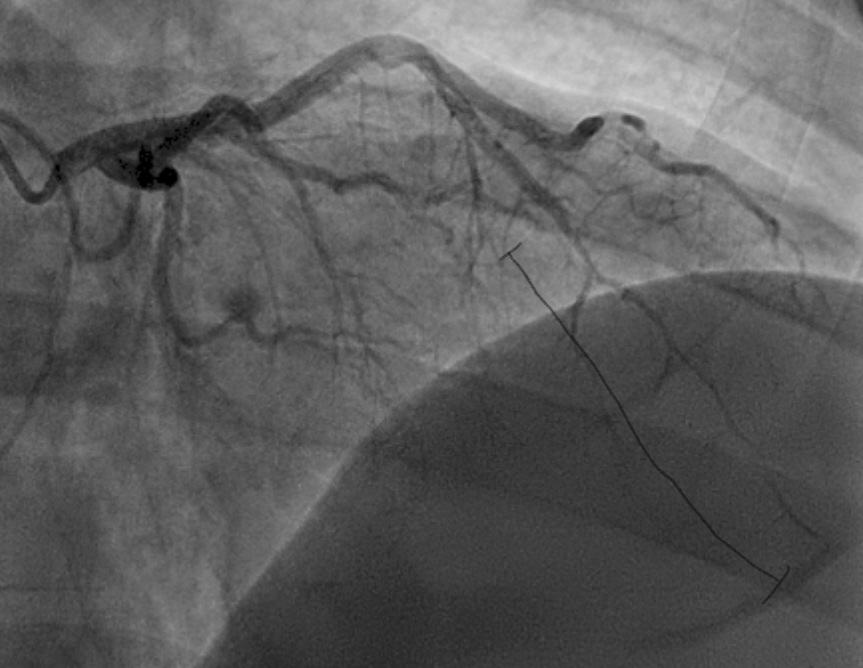

Supplement: Supplementary file 2 [file 11-1-V19-Supp2.jpg]

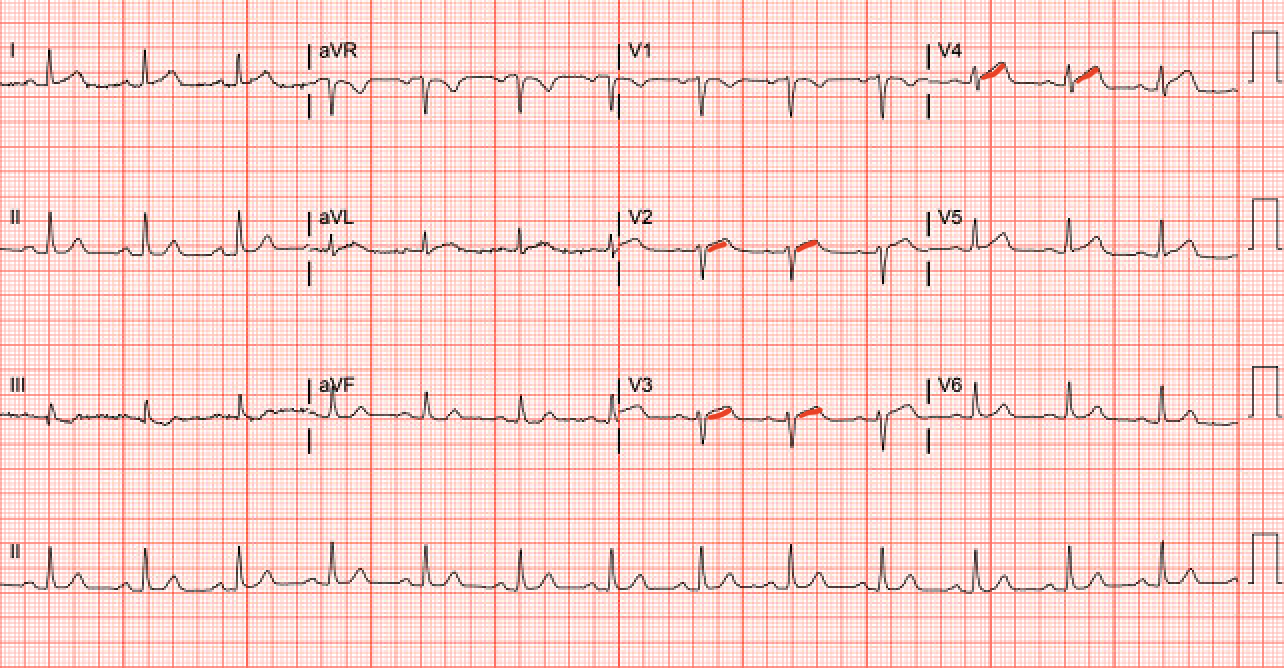

Supplement: Supplementary file 3 [file 11-1-V19-Supp3.jpg]

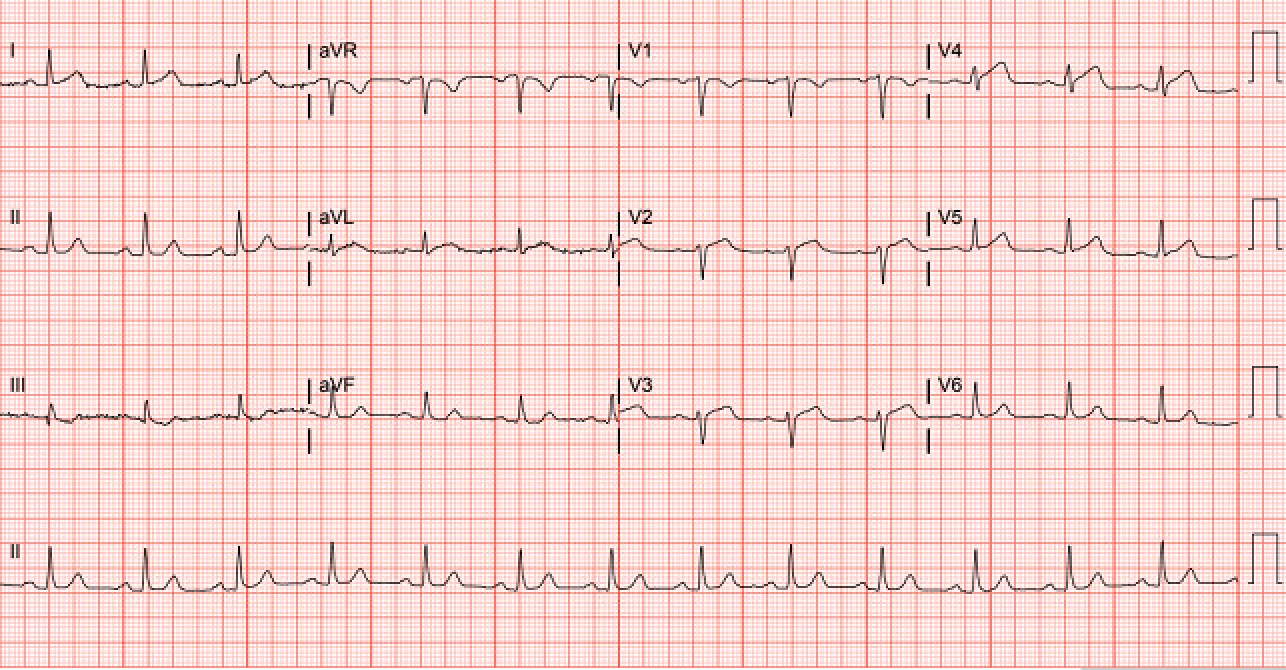

Supplement: Supplementary file 4 [file 11-1-V19-Supp4.jpg]
